# The construction and application of a cell line resistant to novel subgroup avian leukosis virus (ALV-K) infection

**DOI:** 10.1007/s00705-017-3563-2

**Published:** 2017-10-06

**Authors:** Rao Mingzhang, Zhao Zijun, Yuan Lixia, Chen Jian, Feng Min, Zhang Jie, Liao Ming, Cao Weisheng

**Affiliations:** 1 College of Veterinary Medicine, South China Agricultural University, 483 Wushan Road, Tianhe District, Guangzhou, 510642 People’s Republic of China; 2 Key Laboratory of Veterinary Vaccine Innovation of the Ministry of Agriculture, Guangzhou, People’s Republic of China; 3 South China Collaborative Innovation Center for Prevention and Control of Poultry Infectious Diseases and Safety of Poultry Products, Guangzhou, People’s Republic of China; 4 National and Regional Joint Engineering Laboratory for Medicament of Zoonosis Prevention and Control, Guangzhou, People’s Republic of China; 5 Key Laboratory of Zoonosis Prevention and Control of Guangdong Province, Guangzhou, People’s Republic of China

## Abstract

A novel avian leukosis viruses (ALV) subgroup named ALV-K was recently isolated from Chinese indigenous chickens which is different from the subgroups (A to E and J) that have previously been reported to infect chickens. More and more ALV-K strains have recently been isolated from local breeds of Chinese chickens. However, there are no more effective diagnostic methods for ALV-K other than virus isolation followed by envelope gene sequencing and comparison. Viral infection can be blocked through expression of the viral receptor-binding protein. In this study, we have engineered a cell line, DF-1/K, that expresses ALV-K env protein and thereby confers resistance to ALV-K infection. DF-1/K can be used in combination with the ALV-K susceptible cell line DF-1 as a specific diagnostic tool for ALV-K and provides a good tool for further research into the molecular mechanisms of interaction between ALV-K env protein and the host cell receptor.

## Introduction

Avian leukosis viruses (ALVs), which are taxonomically classed as members of the genus *Alpha retrovirus* of the family *Retroviridae*, are divided into 10 subgroups (A to J) based on viral envelope glycoprotein antigenic structure, host range and receptor interference [1]. Members of only six of these subgroups (A-E and J) infect chickens. The main characteristics of avian leukosis (AL), which has resulted in huge economic losses to the poultry industry in China, are serious immunosuppression, growth retardation and tumor-induced mortality. In recent years, a novel ALV subgroup named ALV-K was isolated from indigenous Chinese chickens [2–4]. ALV subgroups are determined based on the gp85 envelope protein [5–7], and based on gp85 amino acid sequence comparisons ALV-K is different from all other known subgroups that infect chickens (A-E and J) [2–4]. Interestingly, several fowl glioma-inducing viruses (FGV) [8–11] reported in Japan and several ALV-K strains (JS11C1, JS11C2, JS11C3, GDFX0601, GDFX0602 and GDFX0603) isolated from native Chinese chicken breeds [2, 3] show over 90 % identity to TW3593 [12] in their gp85 amino acid sequences. Based on phylogenetic analysis of their gp85 genes, these viruses form one large clade that is parallel to other ALV groups. The gp85 gene of TW3593 is different from all other subgroups and this unique ALV is common in Taiwan area [12]. These observations suggest that ALV-K might have existed locally in Chinese chickens for a long time [2–4]. Recently, more and more ALV-K strains have been isolated from local Chinese chicken breeds [13]. These ALV-K isolates replicate more slowly and are less pathogenic than some other ALV strains [4]. The low replication of the ALV-K strain results in a low level of p27 antigen expression, so that chickens infected with exogenous ALV-K are difficult to detect and cause widespread spread, causing interference to ALV control. Therefore, further study of the biological characteristics, genome structure and function of ALV-K, as well as the molecular mechanisms of infection are necessary.

Retroviruses efficiently infect only cells that express specific receptors that interact with the viral envelope glycoproteins [14]. Viral infection and pathogenesis are initiated by the binding of this viral surface protein to its cellular receptor. Indeed infection can be blocked by over expression of the viral receptor-binding protein [15]. Different ALV subgroups use distinct cellular receptors [16]. The ALV envelope protein, a glycoprotein encoded by the virus envelope (env) gene, is the main viral surface protein and determines the virus host range and induces the production of host neutralizing antibodies [16]. Specific interactions between the viral env protein and cell surface receptors are necessary for viral entry into host cells. After being infected with a specific ALV subgroup, viral receptors on the cell surface can be blocked by the viral envelope (env) glycoprotein. These infected cells show superinfection resistance and can specifically resist infection with the same subgroup virus again [16].

Different methods have been established for detecting exogenous ALV, including PCR, real-time PCR, immunofluorescence assay (IFA), traditional virus isolation plus an antigen-capture enzyme-linked immunosorbent assay (ELISA) for group-specific p27 antigen of ALV, as well as loop-mediated isothermal amplification (LAMP), and quantitative competitive reverse transcription PCR (QC-RT-PCR) [17–21]. However, each of these methods has limitations. Some methods were only developed for the detection of ALV-J and ALV-A/B. At present, ALV-K diagnosis relies mainly on virus isolation followed by envelope gene sequencing, and there is lack of other diagnostic methods. For instance, IFA-based diagnostics are based on a specific monoclonal antibody, which is not available for ALV-K. Therefore, IFA cannot be used as a specific diagnostic tool for ALV-K. A genetically engineered cell line resistant to ALV-J infection has been developed and applied to screen large numbers of ALV-J field samples [22] and to efficiently identify chicken Annexin A2 (chANXA2) as a novel receptor for retrovirus ALV-J [15]. In this study we induced super infection resistance to the ALV env protein through stable expression of the ALV-K env protein in DF-1 cells, obtaining a cell line that can resist ALV-K infection. This cell line was used to evaluate clinical plasma samples and investigate the distribution of ALV-K infection in native Chinese chickens. This is the first report of a cell line resistant to infection by a novel ALV subgroup (ALV-K).

## Materials and methods

### Viruses and cell lines

The DF-1 fibroblastic cell line (American Type Culture Collection, Manassas, VA) was used for virus culture. The cells were grown in Dulbecco’s modified Eagle medium (DMEM; GIBCO, USA) supplemented with 10% FBS(Fetal bovine serum, GIBCO, USA) and maintained in DMEM supplemented with 2%FBS at 37 °C in a humidified atmosphere containing 5% CO_2_. The ALV strains GDFX0601 (GenBank accession number KP686142), GDFX0602 (GenBank accession number KP686143) and GDFX0603 (GenBank accession number KP686144) were isolated from a native Chinese yellow feather broiler breeder in southern China in 2014, propagated in DF-1 cells and maintained in our laboratory. The ALV-J subgroup strain CHN06 (GenBank accession number HQ900844) was isolated and identified by our laboratory [23]. Two field ALV-K isolates, GD-147 and GD-179, were isolated from a native Chinese yellow feather broiler breeder in southern China in 2015. The titers of the GDFX0601, GDFX0602, GDFX0603 and CHN06 strains were determined by ELISA and are presented as TCID_50_ml^−1^ calculated using the Reed-Muench method [24].

### Antibodies and reagents

The Flag M2, mouse anti-GFP, mouse Anti-β-actin, and FITC-labeled goat anti-mouse IgG antibodies used in this study were purchased from Sigma (CA, USA). The primary antibodies used for ALV-K detection were single factor anti-sera of ALV-K and were provided by Dr Peng Zhao, Shangdong Agricultural University. Zeocin was purchased from Invitrogen (Shanghai, China). B*am*HI and N*ot*I restriction enzymes and T4 ligase were purchased from New England BioLabs.

### Plasmid construction

The ALV-K env gene was amplified by PCR using the following gene-specific primers: P1: 5’-*CGCGGATCC* GCCACC *ATGGAAGCCGTCATAAAG*GCATTTCTGACTGGATAC-3’and P2:5’-*AAGGAAAAAAGCGGCCGC*TTACACTGCTCCATTTTCG-3’. The primers contain protective bases, restriction enzyme cutting sites (italicized letters), a *Kozak* sequence (underlined letters) and a leader sequence (italicized letters) to improve translational efficiency. PCR was performed in a 50 µl reaction mixture that consisted of template DNA (5µL), 10×PCR buffer (TaKaRa, Dalian, China), 1µM each of forward and reverse primers, 2mM MgCl_2_, 100mM of each deoxynucleoside triphosphate (dNTP), and 1 unit of LA TaqTM DNA Polymerase (TaKaRa, Dalian, China). PCR thermocycling profiles included an initial denaturation for 3 min at 94 °C, followed by 30 cycles of amplification (94 °C for 30 s, 55 °C for 1min and 72 °C for 2 min), as well as a final extension of 72 °C for 8 min. The ALV-K env PCR product was purified using the QIAEX II gel extraction kit (Qiagen, Hilden, Germany), sequenced (Invitrogen, Shanghai, China) and subcloned into the pMD-18T vector (TaKaRa, Dalian, China). The ALV-K env gene was then cloned into the eukaryotic expression vector pcDNA3.1 using the B*am*H I and N*ot* I sites. Three pcDNA-env-K vectors were sequenced and all had the predicted nucleotide sequences. The pcDNA-env-K-flag-EGFP vector, which contains flag and EGFP tags, was constructed by PCR amplification of the EGFP fragment which was ligated with the env gene using N*ot*I and X*ba*I restriction enzyme sites. The fusion fragment was then cloned into the pcDNA3.1 vector.

### Cell transfection and cell screening

The day before transfection, DF-1 cells grown in a monolayer were digested with 0.25% trypsin (GIBCO,USA), and the cells were then adjusted to a density of 1.7 × 10^5^ cells/mL in Dulbecco’s modified Eagle’s medium (GIBCO,USA) with 10% FBS (GIBCO,USA). These were plated in 6-well cell culture plates at 37 °C with 5% CO_2_ until they reached approximately 80% confluence. Transfection of the pcDNA-env-K plasmid, pcDNA-env-K-flag-EGFP plasmid and pcDNA3.1/Zeo(+) plasmid into DF-1 cells was performed using Lipofectamine 3000 (Invitrogen, Shanghai, China) according to the manufacturer’s protocol. The empty plasmid pcDNA3.1/Zeo(+) served as a negative control. After 48 hours, the DF-1 cells grown in monolayer as well as cells in one of the 6-well cell culture plates were digested with 0.25% trypsin (GIBCO, USA), and the cells with the media (DMEM + 15%FBS + 200 µg/mL zeocin) were seeded into 24-well tissue culture plates (500 µL/well). The transfected DF-1 cells were selected for resistance to Zeocin. The following day, cells were treated with 500 µl/well of media containing Zeocin (DMEM+15%FBS+200µg/mL zeocin) and this media was replaced every three days. The Zeocin-resistant cells were passaged for 60 generations and then frozen. After 3 months, these cells were refreshed and cultured in medium free of Zeocin.

### Routine PCR and the real-time PCR assay

Routine PCR tests were carried out with genomic DNA extracted from the ALV-K-resistant cell line, designated as DF-1/K cells, as well as DF-1 cells. The DF-1 cells served as a negative control. The specific primers, reaction mixture, thermocycling profiles were as described above. The PCR product was purified using the Omega Gel Extraction kit (Omega Bio-tek). Total cellular RNA was extracted from DF-1/K cells and DF-1 cells with the RNAfast200 kit (Fastagen, Shanghai, China), followed by cDNA synthesis with the RevertAid First strand cDNA synthesis kit (Fermentas, Canada) according to the manufacturer’s instructions. The cDNA was then used for routine PCR and real-time PCR amplification. Real-time reverse transcription (RT)-PCR was done with primers designed for the envelope gene and gene-specific primers synthesized by TaKaRa Company (Dalian, China): F: 5’-CCCCTGCTATTTAGGCAAGCT-3’, R:5’-AGTTGGCAAGCACCTTGAGAA-3’, Probe:Fam-5’-CCATGTTAGCACCCAACCACACAGAA-3’–Eclips. DNA sequences were determined by Invitrogen (Invitrogen, Shanghai, China). For all reactions, PCR amplification and DNA sequencing were carried out at least twice, independently, to avoid PCR errors. Real-time PCR was performed on an ABI 7500 Real-time PCR System (Applied Bio systems) using Premix ExTaq (Probe qPCR) reagents (TaKaRa, Dalian, China) according to the manufacturer’s specifications. Fluorescent signals were recorded during the elongation step. The β-actin gene served as a reference gene (primers: F: 5’-CCAGCCATGTATGTAGCCATCC-3’, R:5’-CACCATCACCAGAGTCCATCAC-3’, Probe: Fam-5’-CTGTGCTGTCCCTGTATGCCTCTGG -3’–Eclips). The relative expression level of the env gene was normalized to GADPH. Finally, real-time quantitative PCR analysis was carried out using the 2^−ΔΔCT^ method [25].

### Indirect immunofluorescence assay (IFA)

DF-1/K and DF-1 cells were washed with PBS, fixed with cold acetone–alcohol (3:2) for 20 min, washed with PBS again, and then allowed to air-dry. The cells were then incubated with a single factor anti-serum for ALV-K at 37 ℃ for 1 h, washed three times with PBS, and further incubated with goat anti-mouse IgG conjugated with FITC (Sigma, USA) at 37 °C for 1 h. After three washes with PBS, the cells were observed using fluorescence microscopy.

### Western blot analysis

DF-1/K cells and DF-1 cells in 150 mm dishes were harvested by scraping with a rubber policeman and homogenized with NP-40 lysis buffer containing 25 mM Tris, 150 mM NaCl, 1 mM EDTA, 1% NP-40, 5% glycerol (pH 7.4) and a protease inhibitor cocktail (Roche). The lysates were collected and incubated on ice for 10 min. Lysates were cleared by centrifugation at 10,000× g for 5 min at 4 °C. The supernatants were analyzed for total protein content with the BCA protein assay kit (Fermentas, Life Technologies). Total protein (20μg) was resolved by 12% SDS-PAGE and transferred onto nitrocellulose membranes (Whatman, Maidstone, UK). Membranes were blocked with 5% (w/v) skim milk for 1 h at 37 °C, and then incubated overnight at 4 °C with a specific mouse anti-gp85 single factor anti-serum for ALV-K and β-actin (Santa Cruz, sc-1616-R). The β-actin protein served as a reference. After three rinses with PBS Tween20 (PBST) buffer, the membranes were incubated at 37 °C for 1 h with IRDye 800-conjugated anti-mouse IgG secondary antibody (1:10,000; Rockland Immunochemicals, Limerick, PA, USA) diluted in PBS. Membranes were washed three times with PBST, then visualized and analyzed with an Odyssey infrared imaging system (LI-COR Biosciences, Lincoln, NE, USA).

### Immune electron microscopy

The DF-1 and DF-1 / K cells were repaired and sliced with a slice thickness of 75 nm, according to the preparation method for ordinary electron microscopy samples. The ultra-thin slices were transferred to a nickel mesh with Fang Hua film (to ensure slice continuity) and then incubated with goat anti-mouse flag antibodies (Sigma, USA), goat anti-mouse EGFP antibodies (Sigma, USA) and single factor anti-serum for ALV-K (gifts from Dr.P.Zhao, College of Shandong Agricultural University) for 1h at 37 °C. After washing the sections with PBSA solution 6 times, the samples were incubated with 10 nm colloidal gold-labeled goat anti-mouse IgG (Sigma,USA) for 1 h at 37 °C. The sections were washed with ultra-pure water and dried at room temperature. Finally, the samples were examined under a JEM-2010HR transmission electron microscope (JEPL, Japan).

### Antiviral experiment

Five different virus titers ranging from 10^1^ TCID_50_ to 10^5^ TCID_50_ per 0.1 ml of ALV-K (GDFX0601), ALV-A (GD13), ALV-B (CD08) and ALV-J (CHN06) were inoculated per well in 24-well cell culture plates containing 1mL 1.7 × 10^5^cells/well DF-1/K cells, all in triplicate. Three wells on each plate served as negative controls. Each dilution of virus was performed in triplicate. After the inoculum was removed, maintenance medium containing DMEM with 2% FBS was added, and the plates were incubated at 37 °C and 5% CO_2_ for another 6 days. The supernatant fluid was then harvested for ALV p27 antigen ELISA detection (ALV-p27 Ag Test kit, IDEXX, Inc., Westbrook, MA). A mock-infected DF-1 cells group was established in parallel as a control.

## Results

### Cell line screening

**Fig. 1:**
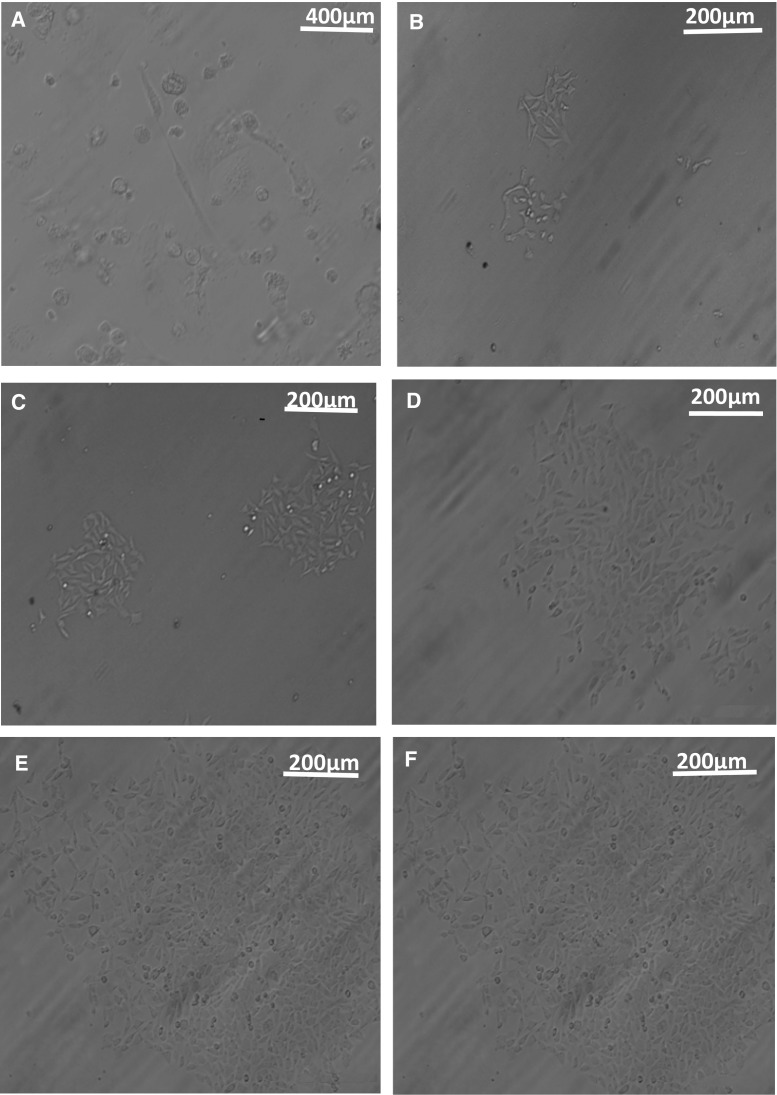
Zeocin selection of cell lines. (**A**) After Zeocin selection, the transfected DF-1 cells formed a single cell colony within 10-15d. The single cell colony appeared to increase in size over the following 6-10d. The differences in the size of the cell clones are shown on days16 (**B**), 18 **(C)**, 19 **(D)** and 20 **(E)**. (**F**) The cells had nearly grown to confluence after approximately 21 days in culture. Magnification is 400× for (A) and 200× for (B-F)

To obtain Zeocin-resistant cells, cells transfected with the pcDNA-env-K plasmid or the pcDNA-env-K-flag-EGFP plasmid were grown in medium containing 200 µg/mL Zeocin. In our culture system, the DF-1 cells formed a single cell colony within 10-15 d (Fig. 1A), and this single cell colony appeared to increase in size over the next 6-10 d (Fig. 1B, C, D, E). The cells grew to near confluence after approximately 21 days in culture (Fig. 1F). After three weeks, the cells were washed with PBS, digested with 0.25% trypsin (Gibco), and then plated in 6-well cell culture plates at 37 °C and 5% CO_2_ until they grew to confluence. The Zeocin-resistant cells were cultured in medium containing Zeocin, passaged continuously for 60 generations and then frozen. After 3 months, these cells were refreshed and cultured in medium free of Zeocin, and the env gene or env protein expression in the transfected DF-1 cell was examined.

### PCR detection of the env gene in DF-1/K cells

**Fig. 2:**
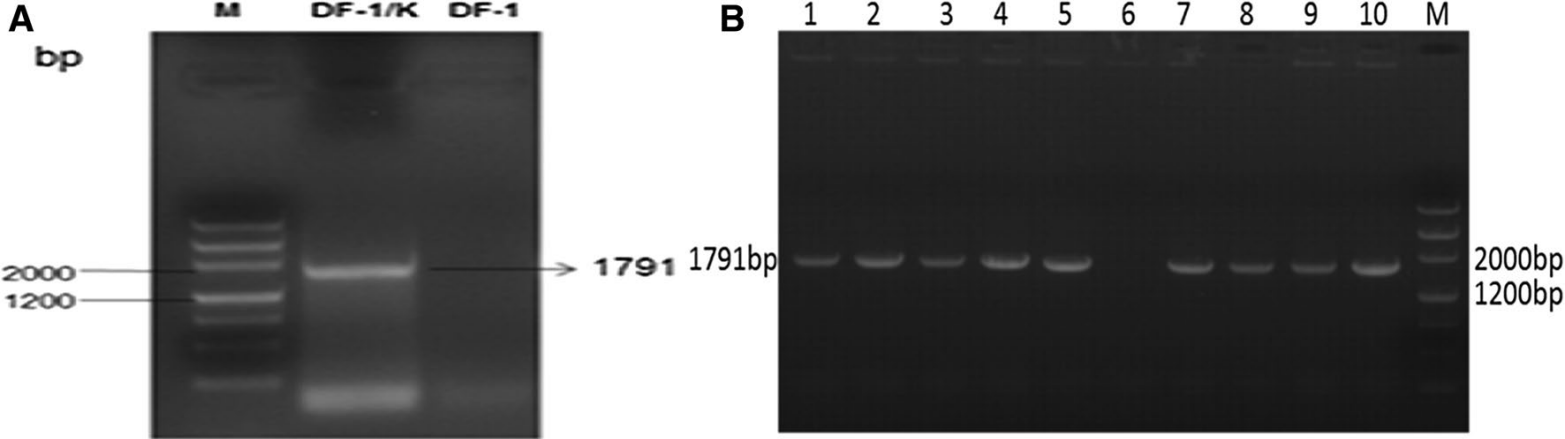
(**A**) PCR amplification of the env gene from DF-1/K cells. (M) DNA marker; (DF-1/K): Genomic DNA extracted from DF-1/K cells; (DF-1): Genomic DNA extracted from DF-1 cells. (**B**) Verification of the stability of the ALV-K env gene in DF-1/K cells during passage. (M) DNAMarker; Lanes 1-5 and 7-10: ALV-K env gene cell-culture passage levels 5, 15, 25, 30, 35, 40, 45, 50, 60, respectively; Lane 6: DF-1 cells (negative control)

PCR was used to detect the ALV-K env gene in the first passage of DF-1/K cells (Fig. 2A) and to confirm ALV-K env gene remained stable in the genome after 20-60 passages (Fig. 2B). A 1791bp amplicon consistent in length with the ALV-K env fragment was amplified from all DF-1/K DNA samples. This PCR product was purified, and sequencing analysis verified that the fragment corresponded to ALV-K env (Fig. 2B). The results demonstrate that the ALV-K env fragment remained genetically stable in DF/K cells during passage.

### Analysis of env gene transcription

**Fig. 3:**
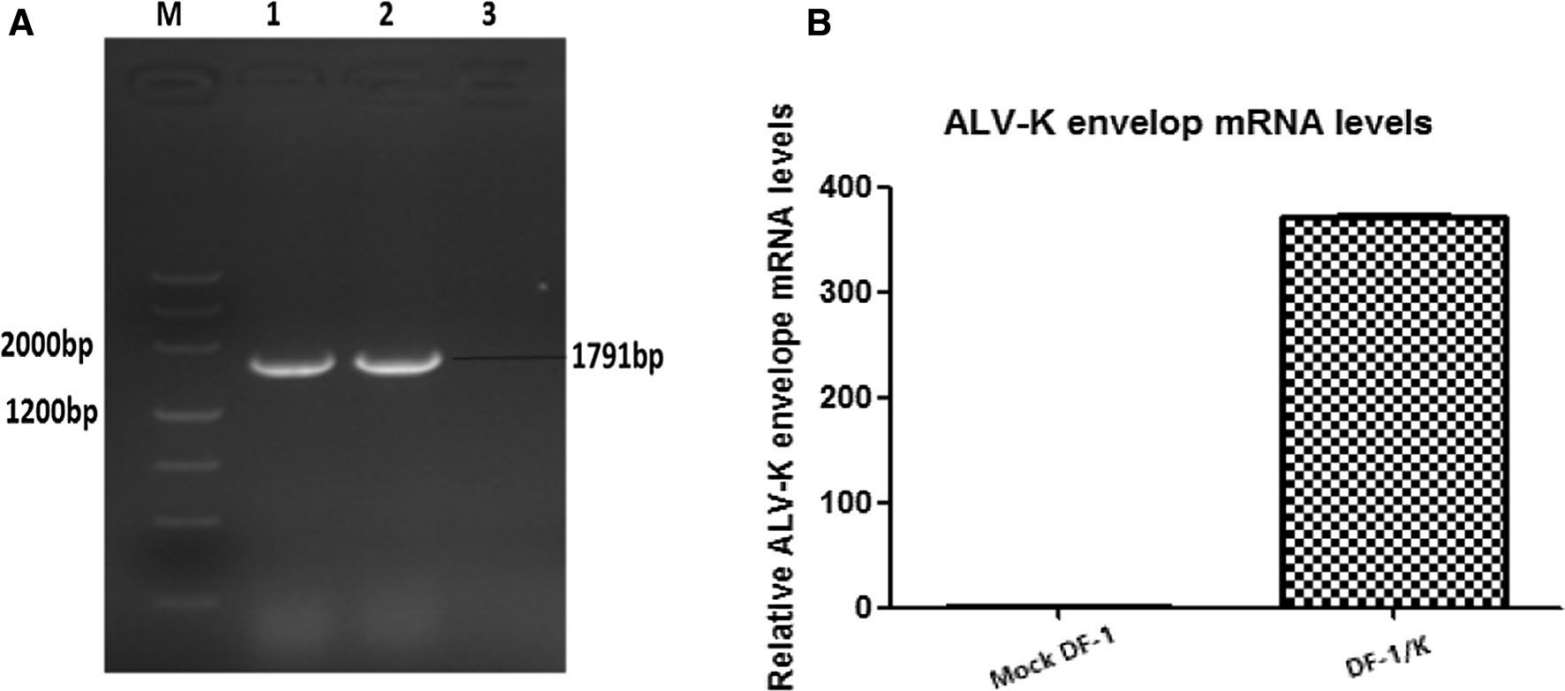
**A)**. PCR amplification of the ALV-K env gene in DF-1/K cells. (M) DNA marker; (1): RNA extracted from DF-1/K cells; (2): Genomic DNA extracted from DF-1/K cells; (3) RNA extracted from DF-1 cells. **B**. Levels of ALV-K env gene transcription in DF-1/K cells were determined by real-time RT-PCR with gene specific primers. DF-1 cells served as a negative control. Data are representative of two independent experiments, both performed in triplicate

Viral envelope gene transcription levels in DF-1/K cells was monitored by routine PCR and real-time RT-PCR with primers designed against the ALV-K env gene. RNA was extracted from DF-1/K and DF-1 cells and reverse transcribed into cDNA, which was then used for routine PCR and real-time PCR amplification. As shown in Fig. 3A, a 1791bp amplicon consistent in length with the ALV-K env fragment was amplified from RNA extracted from DF-1/K cells. Compared to the DF-1 cell negative control, the ALV-K env gene mRNA was highly expressed in DF-1/K cells, but no expression was detected in the DF-1 negative control cells (Fig. 3B).

### Indirect immunofluorescence (IFA) testing of ALV-K env gene expression

**Fig. 4:**
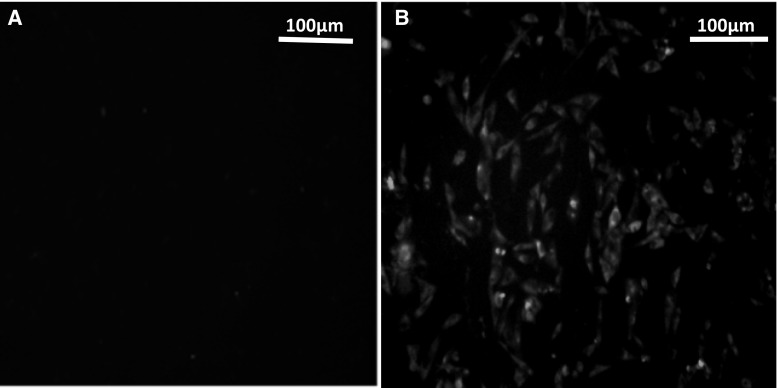
Detection of the ALV-K env protein in DF-1/K cells by IFA. **A**. No green fluorescence was observed in DF-1 cells, which served as negative controls. **B**. Green fluorescence was observed in DF-1/K cells. Magnification is 100x for (**A**) and (**B**)

ALV-K env gene expression was confirmed by detecting the ALV-K env protein using IFA (Fig. 4). The cells were then incubated with a single factor anti-serum for ALV-K, and further incubated with goat anti-mouse IgG conjugated with FITC. No green fluorescence in the cytoplasm could be observed in DF-1 cells, but the green fluorescence signal was bright in DF-1/K cells. This indicates that the exogenous ALV-K env gene was successfully expressed in DF-1/K cells.

### Western blot analysis of env protein expression in DF-1/K cells

**Fig. 5:**
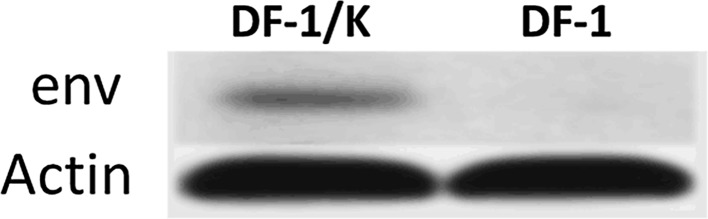
Western blot analysis of env protein expression in DF-1/K cells. (DF-1/K):DF-1/K cell lysate; (DF-1):DF-1 cell lysate was used as a negative control. ALV-K env proteins in the cell lysates were detected with mouse anti-gp85 single factor anti-serum for ALV-K, at a dilution of 1:200. Actin in cell lysates was also detected using actin antibodies to control for equal protein loading. A IRDye 800-conjugated anti-mouse IgG (1:10,000; Rockland Immunochemicals, Limerick, PA, USA) diluted in PBS was used as the secondary antibody

DF-1 and DF-1/K cells grown to monolayer were harvested and lysed. Env proteins within cell lysates were detected by Western blotting using mouse anti-gp85 single factor anti-serum for ALV-K, and IRDye 800-conjugated anti-mouse IgG was used as the secondary antibody. A protein of 90kDa was observed in DF-1/K cells but not DF-1 cells (Fig. 5).

### Antiviral experiment

**Fig. 6:**
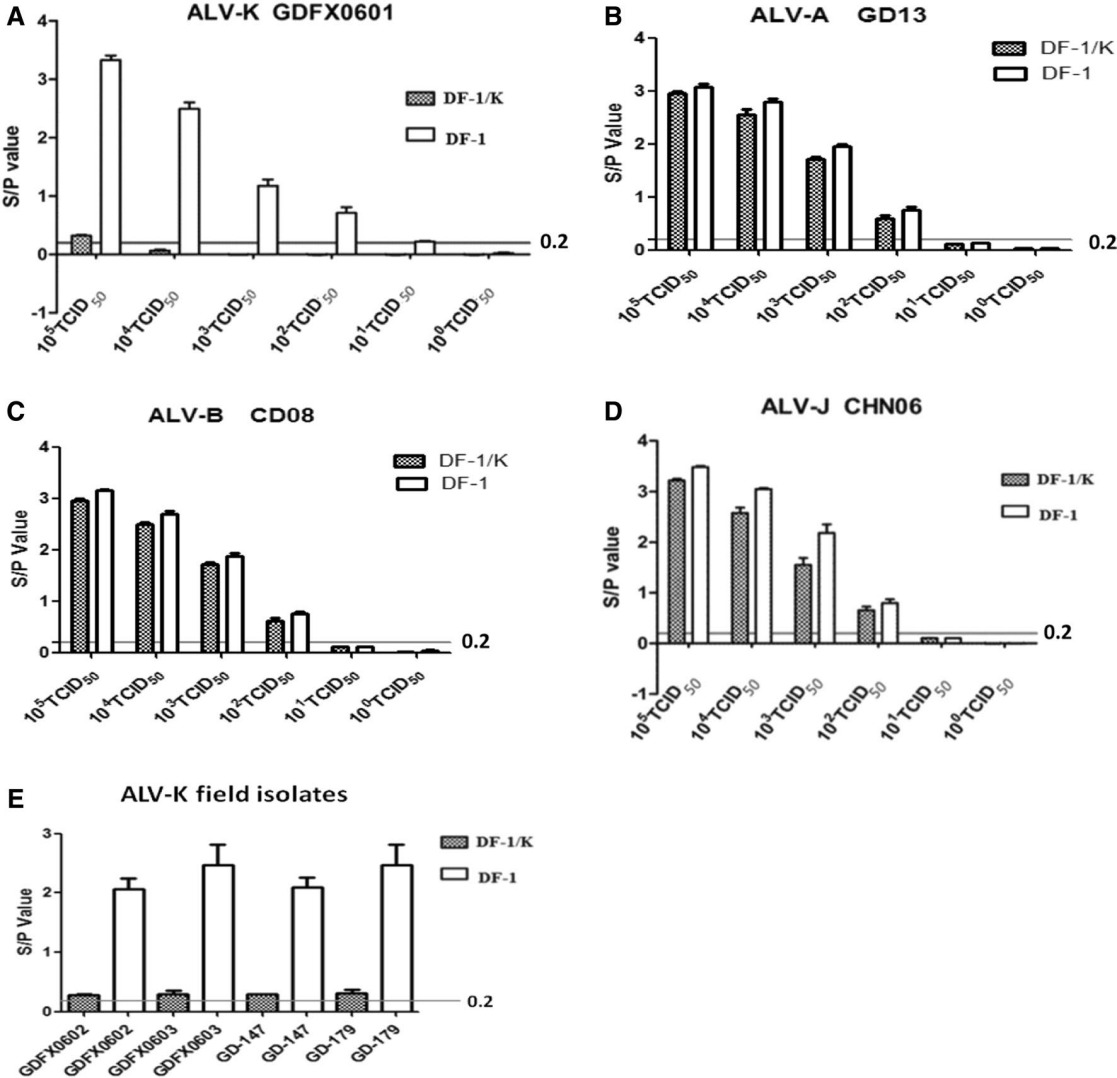
The antiviral experiment results. Five different virus titers from 10^1^ TCID_50_ to 10^5^ TCID_50_ per 0.1 ml of ALV were inoculated per well in 24-well cell culture plates containing 1mL 1.7×10^5^cells/well DF-1/K cells, all in triplicate. **A**. ALV-K (GDFX0601) replication was inhibited in DF-1/K, but not DF-1 cells. **B, C, D**. Replication of ALV-A (GD13), ALV-B (CD08), ALV-J (CHN06) were not inhibited in either DF-1/K and DF-1 cells. **E**. Infection by four ALV-K field isolates was blocked in DF-1/K, but not DF-1cells. In A-E, viral p27 protein levels determined by ELISA are reported. Black lines mean S/P = 0.2

Representative viruses from ALV subgroups A (GD13), B (CD08), J (CHN06) and K (GDFX0601) were used to determine the ability of DF-1/K and DF-1 cells to resist infection. All of the viruses, ALV-A, ALV-B, ALV-J and ALV-K, were capable of infecting and replicating in DF-1 cells. In contrast, only subgroups A, B, J viruses infected the DF-1/K cells, while viruses from subgroup K were largely blocked from infecting these DF-1/K cells. As shown in Fig. 6A~D, DF-1/K cells inhibited the replication of ALV-K but not that of ALV-A, ALV-B, ALV-J viruses based on ELISA measurements of viral p27 protein expression. In order to further determine its antiviral effect on ALV-K, four different field isolates of ALV-K viruses were used to infect DF-1 and DF-1/K cells. All four field isolates were capable of infecting DF-1 cells, but were largely blocked from infecting DF-1/K cells (Fig.6E). The anti-virus assay showed that DF-1/K cells can have resistance to infection at a viral dose of 1×10^4^TCID_50_ ALV-K, and at lower doses infection was completely blocked. When the ALV-K infection dose reached 1 x 10^5^ TCID_50_, the ability of ALV-K to infect DF-1/K cells was still strongly inhibited (Fig. 6A).

### Immune electron microscopy

**Fig. 7:**
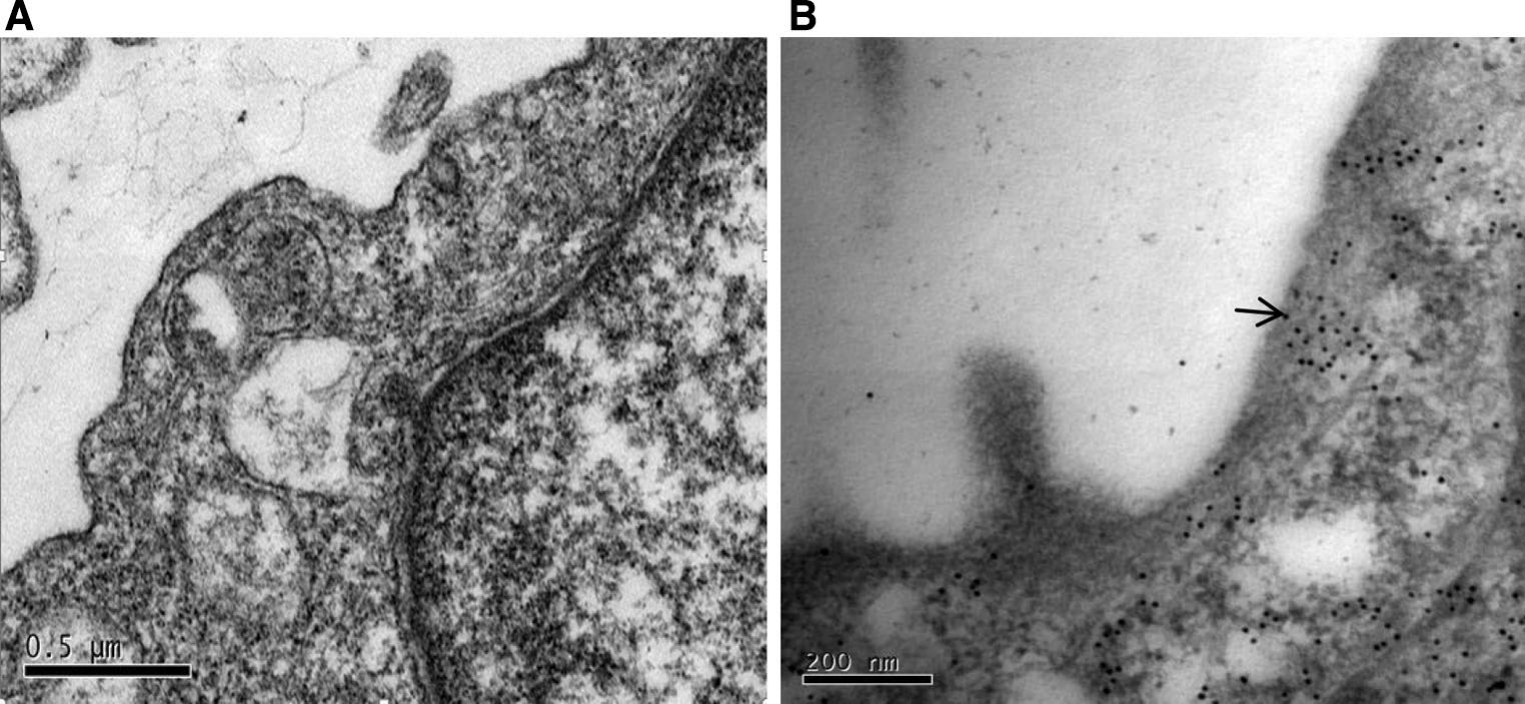
**A)** Electron microscopy image of DF-1/K cells that were pre-incubated with TBS(pH7.4) and subsequently incubated with 10nm colloidal gold-labeled goat anti-mouse IgG. These samples served as negative controls. TEM ×100000. **B**. Electron microscopy image of DF-1/K cells that were pre-incubated with goat anti-mouse flag antibodies, goat anti-mouse EGFP antibodies and single factor anti-serum for ALV-K individually. These were then subsequently incubated with 10nm colloidal gold-labeled goat anti-mouse IgG. TEM ×250000. The arrows point to gold particles

To further understand and monitor the molecular mechanisms underpinning the blocking of cell receptors by membrane-bound envelope glycoproteins, we determined the localization of the ALV-K env fusion protein, which contains flag and EGFP tags, in DF-1/K cells using colloidal gold immune electron microscopy (Fig. 7). The ALV-K fusion protein was detected with goat anti-mouse flag antibodies, goat anti-mouse EGFP antibodies and single factor anti-serum for ALV-K individually. As the secondary antibody, 10nm colloidal gold-labeled goat anti-mouse IgG was used. As a negative control, TBS (pH 7.4) was used in place of the primary antibody of control group. After uranyl acetate and lead citrate staining, images of the samples were obtained with a JEM-2000EX transmission electron microscope. Immunogold particles were observed in the cell membrane. In the controls, there were no immunogold particles in the cell membrane area.

## Discussion

ALVs are RNA viruses that have a high level of genetic variation due to the high error rate of their polymerases and a high recombination rate. They have spread all over the world and caused enormous economic losses in the international poultry industry. ALVs have been divided into 10 different subgroups, subgroup A to J, based on receptor interference patterns, host range, and neutralization by antibodies [26]. So far, most of the ALV in China that has been known to infect chickens belongs to subgroups A, B and J [7, 27–29]. In recent years, a novel ALV subgroup named ALV-K has been isolated from indigenous Chinese chicken breeds, based on gp85 amino acid sequence comparisons [2–4]. Notably, more and more ALV-K strains have been isolated from local Chinese chicken breeds and these isolates represent a new subgroup of ALV viruses that do not induce tumors in SPF chickens and replicate at a relatively slow rate in DF-1 cells [4]. Because the Nationwide Eradication Program (NEP) for ALV in chicken breeder farms was not initiated in China until 2008, ALV infection in chickens has caused serious problems. Over the past decade, many tumor cases induced by ALV have been reported [23, 27]. No effective drugs or vaccines are available for ALV, which is a major challenge for the control and eradication of this virus. The best way to control ALV infection is for all of the exogenous ALV subgroups to be eradicated from chicken breeder flocks. Currently, for large scale identification of these pathogens, a preliminary test is done to see whether ALVs exist and then, if necessary, primers specific for subgroups A, B and J are used to detect the specific ALV subgroup, however this system is not available for ALV-K. At present, ALV-K diagnostic methods mainly rely on virus isolation followed by envelope gene sequencing comparisons, and there is lack of other diagnostic methods.

Retroviruses only efficiently infect cells that express a specific receptor that can interact with their viral envelope glycoproteins [14]. Shortly after the virus has gained entry, it begins to produce viral proteins. As the envelope protein is produced, it saturates the cell surface receptors and blocks superinfection of viruses from the same subgroup [30]. Because saturation of the viral cell receptors of susceptible cells via the expression of viral receptor binding protein can block corresponding viral infection, genetically engineered cell lines resistant to ALV-J infection have been developed [22, 31, 32]. The host range of subgroups A through E is more restricted, and there are lines of chickens that possess genetic resistance to one or more of these subgroups [33–35].

The host range of ALV-K and which chicken lines it is able to infect has not yet been characterized. The lack of genetically resistant cells makes differentiating ALV-K from other subgroups difficult. In addition the lack of genetically resistant chicken lines complicates the classification of field samples. In an attempt to overcome the lack of cell lines that are genetically resistant to ALV-K, we have developed a specific ALV-K-resistant cell line that expresses the subgroup K env protein. The env gene of one ALV-K isolate, GDFX0601, was cloned and expressed in the DF-1 cell line to create the ALV-K-resistant cell line (DF-1/K). This over-expressed env protein should theoretically bind to the cells’ viral receptors and selectively interfere with ALV-K infection. The ALV-K-resistant cell line and its parental cell line DF-1 do not express other ALV proteins and thus the ALV p27 antigen ELISA can be used to monitor ALV infection. To test this system, the env gene in DF-1/K cells was detected by PCR, and the env protein itself was detected by IFA and Western blot. The results showed that DF-1/K cells could stably express the ALV-K env gene. Immune electron microscopy revealed that the ALV-K env fusion protein is localized in the cell membrane area of DF-1/K cells. The anti-virus assay showed that DF-1/K cells showed resistance to ALV-K infection at a viral dose of 1 × 10^4^TCID_50_. ALV-K in field samples can be definitively identified by using ELISA to monitor levels of p27 in DF-1 and DF-1/K cells that have been inoculated with field samples. Only inoculation with ALV-K will result in the detection of p27 in DF-1 cells and no or a lower p27 response in DF-1/K cells. Tests for p27 in cells inoculated with ALV-A, B, J should be positive for both cell types.

In conclusion, we have developed a specific DF-1/K cell line that expresses a viral receptor-binding protein and is resistant only to ALV-K infection. We have not only constructed a cell line that will be a useful diagnostic tool for the novel ALV-K subgroup. Others have demonstrated that cell lines that express viral receptor-binding protein can be efficient tools for isolating functional receptors; a cell line resistant to ALV-J infection was developed and applied to efficiently identify chicken Annexin A2 (chANXA2) as a novel receptor for retrovirus ALV-J [15]. Our DF-1/K cell line will be further applied to identify ALV-K functional receptors and to identify novel anti-viral targets. This study also is helpful to existing efforts to control and eradicate exogenous ALV in local Chinese chickens.

## References

[1] Payne LN, Howes K, Gillespie AM, Smith LM (1992). Host range of Rous sarcoma virus pseudotype RSV (HPRS-103) in 12 avian species: support for a new avian retrovirus envelope subgroup, designated. J Gen Virol.

[2] Wang X, Zhao P, Cui ZZ (2012). Identification of a new subgroup of avian leukosis virus isolated from Chinese indigenous chicken breeds. Bing Du Xue Bao.

[3] Cui N, Su S, Chen Z, Zhao X, Cui Z (2014). Genomic sequence analysis and biological characteristics of a rescued clone of avian leukosis virus strain JS11C1, isolated from indigenous chickens. J Gen Virol.

[4] Li X (2016). Isolation, identification and evolution analysis of a novel subgroup of avian leukosis virus isolated from a local Chinese yellow broiler in South China. Arch Virol.

[5] Zavala G, Cheng S (2006). Detection and characterization of avian leukosis virus in Marek’s disease vaccines. Avian Dis.

[6] Silva RF, Fadly AM, Taylor SP (2007). Development of a polymerase chain reaction to differentiate avian leukosis virus (ALV) subgroups: detection of an ALV contaminant in commercial Marek’s disease vaccines. Avian Dis.

[7] Cui Z, Du Y, Zhang Z, Silva RF (2003). Comparison of Chinese field strains of avian leukosis subgroup J viruses with prototype strain HPRS-103 and United States strains. Avian Dis.

[8] Tomioka Y, Ochiai K, Ohashi K, Kimura T, Umemura T (2003). In vivo infection with an avian leukosis virus causing fowl glioma: viral distribution and pathogenesis. Avian Pathol.

[9] Toyoda T, Ochiai K, Ohashi K, Tomioka Y, Kimura T, Umemura T (2005). Multiple perineuriomas in chicken (Gallus gallus domesticus). Vet Pathol.

[10] Tomioka Y (2004). Genome sequence analysis of the avian retrovirus causing so-called fowl glioma and the promoter activity of the long terminal repeat. J Gen Virol.

[11] Ochi A, Ochiai K, Kobara A, Nakamura S, Hatai H, Handharyani E, Tiemann I, Tanaka IR, Toyoda T, Abe A (2012). Epidemiological study of fowl glioma-inducing virus in chickens in Asia and Germany. Avian Pathol.

[12] Chang SW, Hsu MF, Wang CH (2013). Gene detection, virus isolation, and sequence analysis of avian leukosis viruses in Taiwan country chickens. Avian Dis.

[13] Dong X, Zhao P, Xu B, Fan J, Meng F, Sun P, Ju S, Li Y, Chang S, Shi W, Cui Z (2015). Avian leukosis virus in indigenous chicken breeds. China. Emerg Microbes Infect.

[14] Hunter E, Coffin JM, Hughes SH, Varmus HE (1997). Viral entry and receptors. Retroviruses.

[15] Mei M (2015). Identification of novel viral receptors with cell line expressing viral receptor-binding protein. Scientific Reports..

[16] Holmen SL, Federspiel MJ (2000). Selection of a subgroup A avian leukosis virus [ALV(A)] envelope resistant to soluble ALV(A) surface glycoprotein. Virology.

[17] Kim Y, Brown TP (2004). Development of quantitative competitive-reverse transcriptase-polymerase chain reaction for detection and quantitation of avian leukosis virus subgroup. J Vet Diagn Invest.

[18] Payne LN, Gillespie AM, Howes K (1993). Unsuitability of chicken sera for detection of exogenous ALV by the group-specific antigen ELISA. Vet Rec..

[19] Spencer JL, Gilka F (1982). Lymphoid leukosis: detection of group specific viral antigen in chicken spleens by immunofluorescence and complement fixation. Can J Comp Med.

[20] Zhang X, Liao M, Jiao P, Luo K, Zhang H, Ren T (2010). Development of aloop-mediated isothermal amplification assay for rapid detection of subgroup J avian leukosis virus. J Clin Microbiol.

[21] Dai M, Min F, Di L (2015). Development and application of SYBR Green I real-time PCR assay for the separate detection of subgroup J Avian leukosis virus and multiplex detection of avian leukosis virus subgroups A and B. Virol J.

[22] Hunt HD, Lee LF, Foster D, Silva RF, Fadly AM (1999). A genetically engineered cell line resistant to subgroup J avian leukosis virus infection (C/J). Virology.

[23] Lai HZ, Zhang HN, Ning ZY (2011). Isolation and characterization of emerging subgroup J avian leukosis virus associated with hemangioma in egg-type chickens. Vet Microbiol.

[24] Reed LJ, Muench H (1938). A simple method of estimating fifty percent endpoints. Am J Hyg.

[25] Livak KJ, Schmittgen TD (2001). Analysis of relative gene expression data using real-time quantitative PCR and the 2(-Delta Delta C (T)) Method. Methods.

[26] Weiss RA, Levy JA (1992). Cellular receptors and viral glycoproteins involved in retrovirus entry. The retroviruses.

[27] Cheng Z, Liu J, Cui Z, Zhang L (2010). Tumors associated with avian leukosis virus subgroup J in layer hens during 2007 to 2009 in China. J Vet Med Sci.

[28] Zhang QC, Zhao DM, Guo HJ, Cui ZZ (2010). Isolation and identification of a subgroup A avian leukosis virus from imported meat-type grand-parent chickens. Virol Sin.

[29] Zhang X (2009). Isolation and identification of CD08 strain of avian leukosisvirus associated with hemangioma. Chin J Vet Med.

[30] Crittenden LB (1991). Retroviral elements in the genome of the chicken: Implications for poultry genetics and breeding. Crit Rev Poultry Biol.

[31] Crittenden LB, Salter DW (1992). A transgene, alv6, that expresses the envelope of subgroup A avian leukosis virus reduces the rate of congenital transmission of a field strain of avian leukosis virus. Poultry Sci.

[32] Ye J, Qin A, Shao H (2005). Development of chicken embryo fibroblast cell line resistant to J subgroup avian leukosis virus (ALV-J) infection. Chin J Virol.

[33] Crittenden LB, Stone HA, Reamer RH, Okazaki W (1967). Two loci controlling genetic cellular resistance to avian leukosis–sarcoma viruses. J Virol.

[34] Payne LN, Biggs PM (1964). Differences between highly inbred lines of chickens in the response to Rous sarcoma virus of the chorioallantoic membrane and of embryonic cells in tissue culture. Virology.

[35] Vogt PK, Ishizaki R (1965). Reciprocal patterns of genetic resistance to avian tumor viruses in two lines of chickens. Virology.

